# Multimodal magnetic resonance imaging reveals distinct sensitivity of hippocampal subfields in asymptomatic stage of Alzheimer’s disease

**DOI:** 10.3389/fnagi.2022.901140

**Published:** 2022-08-12

**Authors:** Junjie Wu, Syed S. Shahid, Qixiang Lin, Antoine Hone-Blanchet, Jeremy L. Smith, Benjamin B. Risk, Aditya S. Bisht, David W. Loring, Felicia C. Goldstein, Allan I. Levey, James J. Lah, Deqiang Qiu

**Affiliations:** ^1^Department of Radiology and Imaging Sciences, Emory University School of Medicine, Atlanta, GA, United States; ^2^Department of Neurology, Emory University School of Medicine, Atlanta, GA, United States; ^3^Department of Radiology and Imaging Sciences, Indiana University School of Medicine, Indianapolis, IN, United States; ^4^Athinoula A. Martinos Center for Biomedical Imaging, Department of Radiology, Massachusetts General Hospital, Harvard Medical School, Boston, MA, United States; ^5^Department of Biostatistics and Bioinformatics, Emory University Rollins School of Public Health, Atlanta, GA, United States; ^6^Joint Department of Biomedical Engineering, Emory University and Georgia Institute of Technology, Atlanta, GA, United States

**Keywords:** AD pathology, normal aging, hippocampus subfields, morphometry, functional connectivity, tissue microstructure

## Abstract

While hippocampal atrophy and its regional susceptibility to Alzheimer’s disease (AD) are well reported at late stages of AD, studies of the asymptomatic stage of AD are limited but could elucidate early stage pathophysiology as well as provide predictive biomarkers. In this study, we performed multi-modal magnetic resonance imaging (MRI) to estimate morphometry, functional connectivity, and tissue microstructure of hippocampal subfields in cognitively normal adults including those with asymptomatic AD. High-resolution resting-state functional, diffusion and structural MRI, cerebral spinal fluid (CSF), and neuropsychological evaluations were performed in healthy young adults (HY: *n* = 40) and healthy older adults with negative (HO−: *n* = 47) and positive (HO+ : *n* = 25) CSF biomarkers of AD. Morphometry, functional connectivity, and tissue microstructure were estimated from the structural, functional, and diffusion MRI images, respectively. Our results indicated that normal aging affected morphometry, connectivity, and microstructure in all hippocampal subfields, while the subiculum and CA1-3 demonstrated the greatest sensitivity to asymptomatic AD pathology. Tau, rather than amyloid-β, was closely associated with imaging-derived synaptic and microstructural measures. Microstructural metrics were significantly associated with neuropsychological assessments. These findings suggest that the subiculum and CA1-3 are the most vulnerable in asymptomatic AD and tau level is driving these early changes.

## Introduction

Alzheimer’s disease (AD) is a neurodegenerative disorder characterized by the deposition of amyloid-β (Aβ) plaques and neurofibrillary tangles of phosphorylated tau (P-tau) proteins in the brain (Sperling et al., 2011; Jack et al., 2018). It can take decades for clinical symptoms of cognitive impairment to appear after positive AD pathology (Sperling et al., 2011; Fjell et al., 2014; Jack et al., 2018). With the failure of amyloid reduction therapy to alter the disease trajectory in the symptomatic stages of AD, the field is shifting toward studying early brain changes associated with asymptomatic stage of AD and its transition from healthy aging. In this study, we specifically aimed to employ a multimodal magnetic resonance imaging (MRI) approach to study normal aging and the asymptomatic phase of AD with a focus on subfield vulnerability of the hippocampus. Such studies could shed light on physiological processes leading to asymptomatic AD, which may be different from those at the symptomatic stages (Braak and Del Tredici, 2015; Jagust, 2018), and provide early biomarkers to identify those at high risk of developing cognitive symptoms for patient selection in clinical trials and therapeutic decision-making as effective treatments become available (Dubois et al., 2016).

The hippocampus is one of the earliest brain structures influenced by AD neuropathology (Braak and Braak, 1995; Braak et al., 2006). Hippocampal volume loss (de Flores et al., 2015), disruption of the hippocampal functional network (Dennis and Thompson, 2014; Krajcovicova et al., 2014), and alterations in tissue microstructure measured by diffusion MRI (Chua et al., 2008; Zhang et al., 2014) have been reported in late stages of AD. The hippocampus is heterogeneous and composed of different subfields, including the dentate gyrus (DG), the *cornu ammonis* subregions (CA1–CA4), and the subiculum (Duvernoy, 2005; Iglesias et al., 2015). Histological and structural studies have shown differential vulnerabilities of hippocampal subfields to AD and to normal aging (Small et al., 2011; Maruszak and Thuret, 2014). While most previous imaging findings are based on hippocampal subfield volumetry (de Flores et al., 2015; Chételat, 2018), it is suggested that functional and diffusion MRI can reveal synaptic and microstructural aberrances very early in the AD cascade, before gross anatomical abnormality and cognitive decline (Sperling et al., 2011; Ten Kate et al., 2018). However, controversies remain in regard to hippocampal alterations in the presymptomatic stage (Huijbers et al., 2015; de Flores et al., 2017) and normal aging (de Flores et al., 2015). It is also not clear whether there is a distinct pattern of brain changes between healthy aging and asymptomatic AD. The relative sensitivity of different MR measures to early brain changes associated with asymptomatic AD is also unknown. To answer these questions, we need a study that employs multiple MR sequences, covers a large adult age span and quantifies AD pathology using methods such as cerebrospinal fluid (CSF) assays.

As part of an ongoing study, we performed a cross-sectional analysis that included three groups of cognitively normal adults covering a wide age span: healthy young adults (HY) and healthy older adults with negative (HO−) and positive (HO+) CSF biomarker status of AD. We employed anatomical MRI, high-resolution resting-state functional MRI, and multi-shell diffusion MRI to study alterations in morphometry, intrinsic functional connectivity, and tissue microstructural measures of hippocampal subfields due to normal aging (HY vs. HO−) and AD pathology in the asymptomatic phase (HO− vs. HO+). The imaging metrics were further correlated with CSF biomarkers for AD, including Aβ, total tau (T-tau) and P-tau, as well as an extensive neuropsychological battery assessing memory, language, visuospatial and executive functions.

## Materials and methods

### Participants

A total of 112 cognitively normal participants were included in this study (Table 1), including 40 HY (age = 26.1 ± 3.7 years; 27 females), 47 HO− (see definition below) (age = 65.9 ± 4.8 years; 34 females), and 25 HO+ (age = 69.7 ± 5.6 years; 24 females). HO− and HO+ were part of the Emory Healthy Brain Study (EHBS) (Goetz et al., 2019). The inclusion criteria for the EHBS were self-declared normal cognition with age > 50 years. Exclusion criteria included neurologic and other organic conditions whose natural course or treatment affect cognition, contra-indications to MRI, and factors related to lumbar puncture safety for cognitively normal elderly. This Health Insurance Portability and Accountability Act–compliant study was approved by the Emory University School of Medicine Institutional Review Board. Written informed consent was obtained prior to study participation from all participants in accordance with the Declaration of Helsinki.

**TABLE 1:** Demographics, CSF, and volumetric assessments.

	Groups			HO− vs. HY*^a^*			HO+ vs. HO−*^b^*		
	HY	HO−	Ho+	*t* statistic	*p*-value	(Partial) η^2^	*t* statistic	*p*-value	(Partial) η^2^
*N*	40	47	25						
Age (years)	26.1 ± 3.7	65.9 ± 4.8	69.7 ± 5.6	42.609	<0.001***	0.955	3.012	0.025*	0.115
Sex (female)	27	34	24		1.000			0.079	
Aβ (pg/dl)	NA	571.3 ± 107.2	398.6 ± 118.9	NA			−5.345	<0.001***	0.296
T-tau (pg/dl)	NA	46.1 ± 19.0	99.7 ± 36.1	NA			7.039	<0.001***	0.422
P-tau (pg/dl)	NA	27.7 ± 10.5	46.0 ± 21.5	NA			4.411	<0.001***	0.223
T-tau/Aβ ratio	NA	0.081 ± 0.029	0.263 ± 0.105	NA			9.598	<0.001***	0.575
eTIV (mm^3^)	1.54 × 10^6^ ± 1.68 × 10^5^	1.49 × 10^6^ ± 1.50 × 10^5^	1.53 × 10^6^ ± 1.13 × 10^5^	−1.585	0.467	0.029	2.956	0.026*	0.114
Whole hippocampal volume (mm^3^)	8.28 × 10^3^ ± 7.62 × 10^2^	7.79 × 10^3^ ± 7.95 × 10^2^	7.57 × 10^3^ ± 8.57 × 10^2^	−2.470	0.078	0.068	−1.248	0.216	0.023
Parahippocampal volume (mm^3^)	4.40 × 10^3^ ± 5.10 × 10^2^	3.99 × 10^3^ ± 4.37 × 10^2^	3.81 × 10^3^ ± 4.16 × 10^2^	−3.552	0.004**	0.132	−1.911	0.175	0.052
ERC volume (mm^3^)	4.15 × 10^3^ ± 1.02 × 10^3^	4.00 × 10^3^ ± 6.28 × 10^2^	3.66 × 10^3^ ± 5.61 × 10^2^	−0.359	1.000	0.002	−2.068	0.170	0.060
PRC volume (mm^3^)	3.93 × 10^3^ ± 6.80 × 10^2^	3.75 × 10^3^ ± 4.50 × 10^2^	3.54 × 10^3^ ± 4.07 × 10^2^	−0.896	1.000	0.010	−1.927	0.175	0.052

HY, healthy young adults; HO−, healthy older adults with negative CSF biomarker status; Ho+, healthy older adults with positive CSF biomarker status; Aβ, Amyloid-β 1-42; T-tau, total tau; P-tau, tau phosphorylated at threonine 181; eTIV, estimated total intracranial volume; ERC, entorhinal cortex; PRC, perirhinal cortex; NA, not applicable.

^a^Group differences between HO− and HY were evaluated using an independent t-test (for age), a chi-square test (for sex), general linear models (GLM) with sex as a covariate (for eTIV), or GLM with sex and eTIV as covariates (for regional volumes).

^b^Group differences between Ho+ and HO− were evaluated using an independent t-test (for age), a chi-square test (for sex), GLM with age and sex as covariates (for CSF assessments and eTIV), or GLM with age, sex and eTIV as covariates (for regional volumes).

Significant at *P < 0.05, **P < 0.01 and ***P < 0.001, Holm-Bonferroni corrected.

### Magnetic resonance imaging acquisition

MRI data were acquired on a Siemens Magnetom Prisma 3-Tesla scanner (Siemens Healthcare, Erlangen, Germany) equipped with a 32-channel head array coil. T_1_-weighted anatomical images were acquired using a magnetization-prepared rapid acquisition with gradient echo (MPRAGE) sequence (TR/TE = 2,300/2.96 ms, TI = 900 ms, FA = 9°, voxel size = 1 × 1 × 1 mm^3^, 208 slices). A 10-min resting-state functional MRI was performed using a multiband accelerated gradient-echo echo-planar imaging sequence (TR/TE = 1,890/30 ms, FA = 52°, voxel size = 1.5 × 1.5 × 1.5 mm^3^, 81 slices, multiband factor = 3, and 320 volumes). Diffusion MRI images were acquired using a multiband accelerated spin-echo echo-planar sequence (TR/TE = 2,600/80 ms, FA = 90°, voxel size = 2 × 2 × 2 mm^3^, 69 slices, multiband factor = 3, diffusion gradient duration = 24.74 ms, diffusion time = 39.78 ms). A multi-shell diffusion-weighting scheme was used with 3 b_0_ volumes, 10 diffusion weighting directions for b = 150 s/mm^2^, 10 directions for b = 350 s/mm^2^, 64 directions for b = 1,000 s/mm^2^, 64 directions for b = 2,000 s/mm^2^, 64 directions for b = 3,000 s/mm^2^ and 104 directions for b = 5,000 s/mm^2^. Five b_0_ volumes were also acquired with reversed phase-encoding polarity and used to perform correction for susceptibility-induced image distortions.

### Magnetic resonance imaging analysis

Image segmentation on T_1_-weighted MPRAGE images was performed using FreeSurfer 6.0 (Athinoula A. Martinos Center for Biomedical Imaging, Harvard University, Boston, Massachusetts, United States). Hippocampal subfield ROIs including the subiculum, CA1-3, and CA4-DG, as well as medial temporal lobe ROIs including parahippocampus, entorhinal cortex (ERC), and perirhinal cortex (PRC) were extracted from the results. Similar to the strategy adopted by previous studies (Kulaga-Yoskovitz et al., 2015; Vos de Wael et al., 2018), CA4 and DG were merged since the CA4 lies within the DG in hippocampal subfield atlas of FreeSurfer (Iglesias et al., 2015), and CA1, CA2 and CA3 were combined owing to a lack of distinguishable contrast between them. Estimated total intracranial volume (eTIV) and volumes of whole hippocampus, parahippocampus, ERC, and PRC were also calculated from the FreeSurfer segmentation. The volumes of bilateral ROIs were combined and normalized to eTIV. To study morphometric changes, we performed shape analysis of the hippocampus using FSL FIRST (Patenaude et al., 2011). Instead of performing direct segmentation of hippocampal subfields, FSL FIRST outputs parameterized surface meshes to provide a direct and local measure of geometric change, followed by the mapping of any detected group differences to anatomical regions.

**FIGURE 1:**
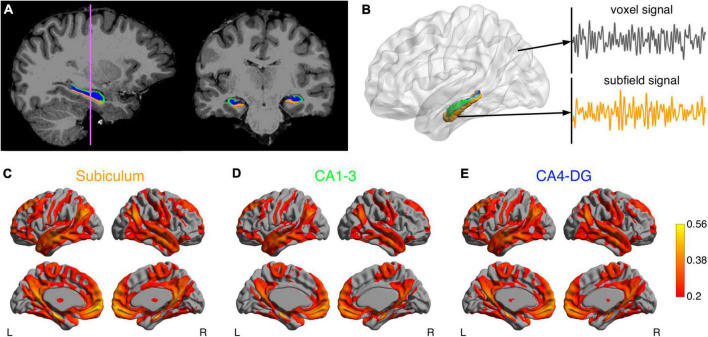
Analysis pipeline of the hippocampal subfield networks. **(A)** Segmentations of the subiculum (orange), CA1-3 (green) and CA4-DG (blue) superimposed on T_1_-weighted anatomical image. **(B)** After data preprocessing, the functional MRI time course was extracted for each voxel. The mean time course of each subfield was also computed as seed signal. Connectivity maps for the subiculum **(C)** CA1-3 **(D)** and CA4-DG **(E)** networks averaged across a group of healthy young adults.

Figure 1 illustrates the procedure for functional connectivity analysis of the hippocampal subfields, which is similar to a previously published pipeline (Vos de Wael et al., 2018). Preprocessing of functional MRI images was performed using FSL 6.0 (Wellcome Centre for Integrative Neuroimaging, University of Oxford, Oxford, United Kingdom). The functional images were corrected for B_0_ field inhomogeneity and head motion. Noise was then removed using the ICA-FIX procedure (Salimi-Khorshidi et al., 2014). Denoised images were bandpass-filtered to retain signal components with temporal frequency between 0.01 and 0.1 Hz. The resulting images were warped to T_1_-weighted images using a combination of boundary-based and rigid body registrations. The transformations were concatenated with the non-linear transformation from native T_1_-weighted images to the Montreal Neurological Institute (MNI) ICBM152 stereotaxic space, to warp functional MRI images to the MNI ICBM152 space.

The functional networks were identified by using a seed-based functional connectivity analysis method on the functional MRI data. As a high-level strategy, we smoothed signals from target brain regions to improve SNR, but did not smooth signals from seed regions to minimize partial volume effects. Specifically, given that the seed ROIs were relatively small, the average temporal signal in each seed ROI of the preprocessed images was calculated but not spatially smoothed to minimize the partial volume effects. The target brain signals were spatially smoothed with a 6 mm full-width-at-half-maximum Gaussian kernel. Pearson correlation coefficients were then calculated between the unsmoothed temporal signals in the seed regions and the smoothed signal in each target voxel throughout the brain. The resulting correlation coefficients were Fisher *z*-transformed.

The raw diffusion-weighted images were processed using MRtrix3 (Brain Research Institute, Melbourne, Australia) to reduce signal noise, effects from Gibbs ringing, subject movement, susceptibility induced image distortions, and B_1_ field inhomogeneity. Microstructural parameters including neurite density index (NDI), orientation dispersion index (ODI), and volume fraction of isotropic water diffusion (V_*iso*_) were calculated according to neurite orientation dispersion and density imaging (NODDI) model (Zhang et al., 2012) using Dmipy toolbox.^1^ NDI evaluates the volume fraction of neurites with the values ranging from 0 (no intraneurite diffusion) to 1 (complete intraneurite diffusion). ODI evaluates the degree of neurite dispersion with the values ranging from 0 (no dispersion) to 1 (full dispersion). V_*iso*_ evaluates the volume fraction of freely moving water, such as CSF, with the values ranging from 0 (no CSF-like fluid) to 1 (complete CSF-like fluid). The NODDI measures of ROIs were extracted according to a procedure proposed in Nazeri et al. (2017). Briefly, for each subject, a pseudo-T_1_ image in diffusion space (Nazeri et al., 2015) was generated and linearly registered to subject T_1_ space. The inverse transform matrix was then applied to transform FreeSurfer-generated ROIs to individual diffusion space. Due to 2 mm^3^ spatial resolution in diffusion MRI, the regional robust mean values were calculated by excluding top and bottom 5% data as extreme values. Since we found that imaging metrics were different between subfields, excluding the extreme values could potentially reduce the effects of partial volume.

### Cerebral spinal fluid sampling and analysis

Lumbar punctures were performed in the cognitively normal elderly at the L3–4 or L4–5 interspace with a 24-g Sprotte atraumatic spinal needle. CSF was collected into polypropylene transfer tubes and frozen on dry ice within 1 h after collection. Aliquots (0.5 mL) were prepared from these samples after thawing (1 h) at room temperature and gentle mixing. Following a single freeze-thaw cycle, amyloid-β 1–42, total tau, and tau phosphorylated at threonine 181 were measured using the multiplex xMAP Luminex platform (Luminex Corp., Austin, Texas, United States) with Innogenetics (INNO-BIA AlzBio3; Ghent, Belgium) immunoassay kit-based reagents. All assays were performed in a single laboratory (AKESOgen, Peachtree Corners, Georgia, United States). Subsequent aliquots were stored in bar code-labeled FluidX 0.9 ml polypropylene vials (Brooks Life Sciences, Chelmsford, Massachusetts, United States) at −80^°^C. T-tau/Aβ ratio was calculated as an indicator of Aβ and tau burden. Local cutoffs for AD-biomarker positivity were established by analyzing results from 1,298 individuals including those diagnosed with mild cognitive impairment (MCI) or dementia due to AD (*n* = 345), other non-AD dementia (*n* = 37), and normal controls (*n* = 916). Latent class analysis with adjustments for assay batch effects showed optimum model fit for a three-class solution, and we selected a cutoff value of T-tau/Aβ ratio of 0.165 (sensitivity = 94.3%, specificity = 87.8%) to classify Ho+ individuals in the EHBS cohort.

### Neuropsychological assessments

A standardized neuropsychological test battery was administered, including the Montreal Cognitive Assessment (MoCA) for overall cognitive status, Free and Cued Selective Reminding Test (FCSRT) for verbal episodic memory, Rey Complex Figure Test (RCFT) for visual memory and visuospatial functioning, Judgment of Line Orientation (JoLO) for visuospatial ability, Letter Fluency (FAS) for language and executive functioning, Animal Fluency for language and semantic memory, Trail Making Test Part A (TMTA) for processing speed, and Trail Making Test Part B (TMTB) for executive functioning.

### Statistical analysis

Functional connectivity and diffusion metrics between hippocampal subfields were compared using paired *t*-tests in the HY.

Effects of normal aging and asymptomatic AD pathology were evaluated by comparing HO− with HY, and HO+ with HO−, respectively. Age between groups was compared using an independent *t*-test. Sex distribution between groups was compared using a chi-square test. The CSF, volumetric, and neuropsychological assessments between groups were compared using general linear models with sex as a covariate for HY vs. HO−, and with age and sex as covariates for HO− vs. HO+. To study the effects of normal aging, group differences in functional connectivity and diffusion metrics between HY and HO− were examined using general linear models with sex, normalized whole hippocampal volume and eTIV as covariates. Besides eTIV, we additionally included normalized hippocampal volume as a covariate to investigate whether metrics of hippocampal subfields provided additional values that were not accounted for by whole hippocampal volume. We fit a separate model for each of the following outcome variables: mean functional connectivity of the subiculum, the CA1-3 and the CA4-DG networks, as well as diffusion metrics of the hippocampal subfields. A parallel analysis with age, sex, normalized hippocampal volume and eTIV as covariates was performed to compare HO− and Ho+ groups to evaluate influences of AD pathology.

Associations of functional connectivity and diffusion MRI measures with neuropsychological performance and CSF biomarker measurements were estimated using pairwise partial correlation with age, sex, normalized hippocampal volume, and eTIV as covariates. The statistical analyses used a two-tailed level of 0.05 for defining statistical significance, and Holm-Bonferroni correction was applied to adjust for multiple comparisons.

In addition to ROI-based analysis of functional connectivity, voxel-wise analysis was carried out to further reveal which brain regions in the subfield networks showed significant differences/correlations. Permutation-based statistical analyses (5,000 iterations) were performed with threshold-free cluster enhancement (TFCE) where family-wise error (FWE) rate was controlled at 0.05.

## Results

### Hippocampal subfield networks and diffusion metrics

The mean hippocampal subfield networks across the 40 HY are illustrated in Figures 1C–E. These three networks were visually similar to each other, which largely overlapped with the default mode, executive control, sensorimotor, and temporolimbic networks, in line with previous hippocampal subfield connectivity analyses (Vos de Wael et al., 2018). However, some differences were observed between the networks (Supplementary Figure 1): the subiculum showed higher connectivity with most brain regions in the hippocampal network and lower connectivity with left temporal pole and left posterior temporal fusiform cortex, compared to CA1-3; CA4-DG showed higher connectivity with precuneus, cuneus, and left frontal pole, relative to CA1-3. Moreover, significant differences in microstructural metrics were found between hippocampal subfields (Supplementary Table 1). These differences suggested that despite partial volume effects, subfield-based quantifications did provide more information than what was provided by whole hippocampus-based quantifications.

### Effects of normal aging

Comparison of neuropsychological assessments (Table 2) showed HO− performed significantly worse than HY on the MoCA [*t*(69) = −3.064, *P* = 0.024, Holm-Bonferroni corrected, partial η^2^ = 0.120], FCSRT free recall [*t*(67) = −3.705, *P* = 0.004, Holm-Bonferroni corrected, partial η^2^ = 0.170], RCFT immediate free recall [*t*(66) = −4.466, *P* < 0.001, Holm-Bonferroni corrected, partial η^2^ = 0.232], and RCFT delayed free recall [*t*(66) = −4.557, *P* < 0.001, Holm-Bonferroni corrected, partial η^2^ = 0.239].

**TABLE 2:** Neuropsychological assessments.

	Groups			HO− vs. HY*^a^*			Ho+ vs. HO−*^b^*		
	HY	HO−	Ho+	*t* statistic	*p*-value	Partial η^2^	*t* statistic	*p*-value	Partial η^2^
MoCA	28.1 ± 1.9	26.4 ± 2.5	25.5 ± 2.4	–3.064	0.024*	0.120	–1.034	1.000	0.016
FCSRT free recall	39.7 ± 4.0	36.0 ± 4.1	35.4 ± 3.3	–3.705	0.004**	0.170	–0.596	1.000	0.006
RCFT immediate free recall	24.8 ± 5.4	17.8 ± 6.5	16.1 ± 5.2	–4.466	< 0.001***	0.232	–0.408	1.000	0.003
RCFT delayed free recall	24.3 ± 5.7	16.6 ± 6.9	14.6 ± 6.0	–4.557	< 0.001***	0.239	–0.220	0.826	0.001
RCFT copy accuracy score	33.8 ± 2.5	32.3 ± 2.7	30.6 ± 4.1	–2.071	0.252	0.061	–0.561	1.000	0.005
JoLO	25.0 ± 2.8	25.1 ± 4.0	23.1 ± 4.0	0.288	0.774	0.001	–0.274	1.000	0.001
Letter fluency (FAS)	46.4 ± 6.8	40.9 ± 10.8	43.3 ± 12.7	–2.264	0.189	0.070	1.264	1.000	0.024
Animal fluency	24.2 ± 5.5	21.8 ± 4.7	21.7 ± 4.8	–1.848	0.276	0.048	–0.797	1.000	0.010
TMTA	28.3 ± 11.6	34.4 ± 12.4	35.4 ± 10.6	2.057	0.215	0.058	0.337	1.000	0.002
TMTB	62.7 ± 29.9	81.0 ± 45.3	86.5 ± 31.0	1.818	0.219	0.046	0.443	1.000	0.003
TMTB-TMTA	34.4 ± 24.0	46.6 ± 44.0	51.1 ± 24.6	1.273	0.414	0.023	0.582	1.000	0.005

HY, healthy young adults; HO−, healthy older adults with negative CSF biomarker status; Ho+, healthy older adults with positive CSF biomarker status; MoCA, Montreal Cognitive Assessment; FCSRT, Free and Cued Selective Reminding Test; RCFT, Rey Complex Figure Test; JoLO, Judgment of Line Orientation; TMTA, Trail Making Test Part A; TMTB, Trail Making Test Part B.

^a^Group differences between HO− and HY were evaluated using general linear models with sex as a covariate.

^b^Group differences between Ho+ and HO− were evaluated using general linear models with age and sex as covariates.

Significant at *P < 0.05, **P < 0.01 and ***P < 0.001, Holm-Bonferroni corrected.

**FIGURE 2:**
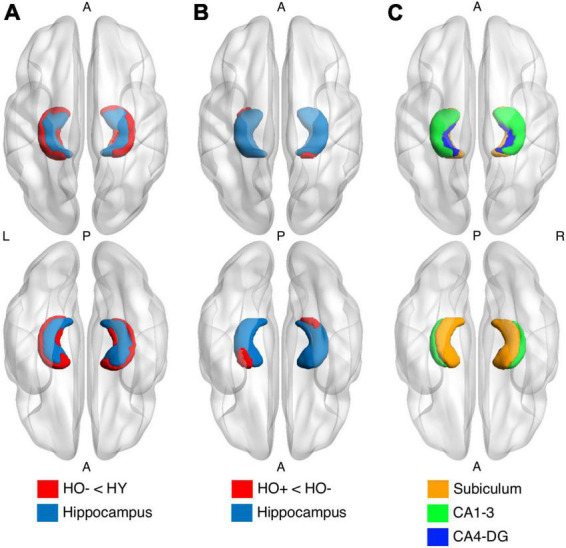
Shape analysis in the hippocampus (light blue) using FSL FIRST shows group differences between healthy young adults (HY) and healthy older adults with negative CSF biomarker status (HO−) **(A)** and between HO− and healthy older adults with positive CSF biomarker status (Ho+) **(B)**. HO− < HY and Ho+ < HO− (red) were found. *P*-values were determined using general linear models with age and sex as covariates. The results were corrected using threshold-free cluster enhancement where family-wise error rate was controlled at 0.05. **(C)** Segmentations of the subiculum (orange), CA1-3 (green) and CA4-DG (blue).

HO− showed significantly reduced parahippocampal volume [*t*(83) = −3.552, *P* = 0.004, Holm-Bonferroni corrected, partial η^2^ = 0.132] and a trend toward lower whole hippocampal volume [*t*(83) = −2.470, *P* = 0.078, Holm-Bonferroni corrected, partial η^2^ = 0.068] as compared to HY (Table 1). Shape analysis using FSL FIRST showed that compared to HY, regional atrophy (*P* < 0.05, FWE corrected using TFCE) was found in HO− in all three hippocampal subfields, especially CA1-3 (Figures 2A,C).

**FIGURE 3:**
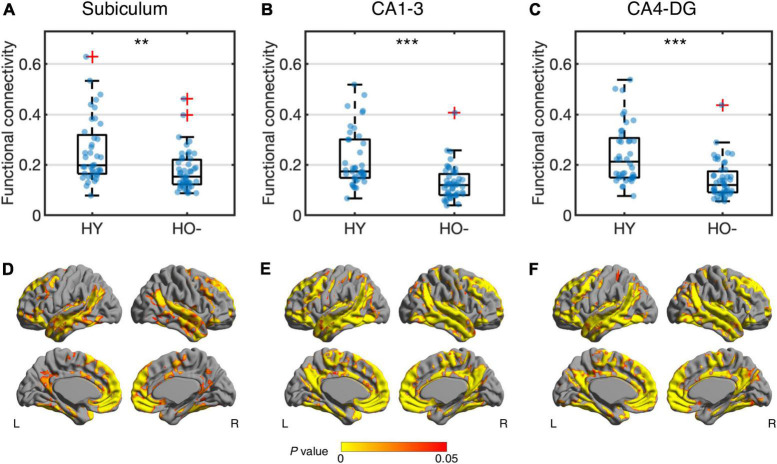
Group differences of functional connectivity in the subiculum **(A,D)**, CA1-3 **(B,E)** and CA4-DG **(C,F)** networks between healthy young adults (HY) and healthy older adults with negative CSF biomarker status (HO−). Box plots show the median, quartiles and whiskers that represent 1.5 × the interquartile range. *P*-values were determined using general linear models with sex, normalized whole hippocampal volume, and total intracranial volume as covariates, and adjusted for multiple comparisons using Holm-Bonferroni correction. In voxel-wise analysis **(D–F)**, the results were corrected using threshold-free cluster enhancement where family-wise error rate was controlled at 0.05. Significant at **P < 0.01 and ***P < 0.001.

**FIGURE 4:**
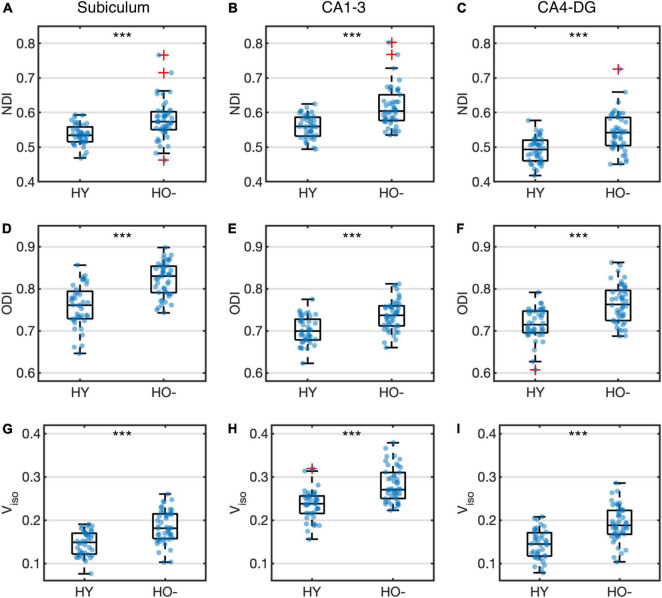
Group differences of neurite density index (NDI) **(A–C)**, orientation dispersion index (ODI) **(D–F)** and volume fraction of isotropic water diffusion (V_*iso*_) **(G–I)** in the subiculum **(A,D,G)**, CA1-3 **(B,E,H)** and CA4-DG **(C,F,I)** between healthy young adults (HY) and healthy older adults with negative CSF biomarker status (HO−). Box plots show the median, quartiles and whiskers that represent 1.5 × the interquartile range. *P*-values were determined using general linear models with sex, normalized whole hippocampal volume, and total intracranial volume as covariates, and adjusted for multiple comparisons using Holm-Bonferroni correction. Significant at ***P < 0.001.

Compared to HY, HO− showed decreased functional connectivity (Figure 3) and increased diffusion MRI measures (Figure 4) in all three hippocampal subfields. Hippocampal subfield functional connectivity was lower in HO− compared to HY (Figures 3A–C), including the subiculum [*t*(82) = −2.960, *P* = 0.004, Holm-Bonferroni corrected, partial η^2^ = 0.097], CA1-3 [*t*(82) = −4.191, *P* < 0.001, Holm-Bonferroni corrected, partial η^2^ = 0.176], and CA4-DG [*t*(82) = −4.165, *P* < 0.001, Holm-Bonferroni corrected, partial η^2^ = 0.175] networks. Voxel-wise analysis (Figures 3D–F) showed widespread differences in most regions of the hippocampal network (*P* < 0.05, FWE corrected using TFCE). For diffusion metrics (Figure 4), HO− showed an increase in NDI [the subiculum: *t*(82) = 4.047, partial η^2^ = 0.168; CA1-3: *t*(82) = 4.670, partial η^2^ = 0.212; CA4-DG: *t*(82) = 4.542, partial η^2^ = 0.203], ODI [the subiculum: *t*(82) = 6.010, partial η^2^ = 0.308; CA1-3: *t*(82) = 4.182, partial η^2^ = 0.178; CA4-DG: *t*(82) = 4.736, partial η^2^ = 0.217], and V_*iso*_ [the subiculum: *t*(82) = 4.824, partial η^2^ = 0.223; CA1-3: *t*(82) = 4.950, partial η^2^ = 0.232; CA4-DG: *t*(82) = 5.090, partial η^2^ = 0.242] in all three hippocampal subfields (all *P* < 0.001, Holm-Bonferroni corrected) as compared to HY.

Compared to HY, HO− showed lower functional connectivity (Supplementary Figure 2) and higher diffusion MRI metrics (Supplementary Figure 3) in medial temporal lobe regions. Decreased functional connectivity was observed in the whole hippocampus [*t*(82) = −3.632, *P* = 0.004, Holm-Bonferroni corrected, partial η^2^ = 0.139], parahippocampus [*t*(82) = −2.952, *P* = 0.016, Holm-Bonferroni corrected, partial η^2^ = 0.096], ERC [*t*(82) = −3.095, *P* = 0.013, Holm-Bonferroni corrected, partial η^2^ = 0.105], and PRC [*t*(82) = −3.655, *P* = 0.004, Holm-Bonferroni corrected, partial η^2^ = 0.140] in HO− as compared to HY (Supplementary Figure 2). For diffusion measures (Supplementary Figure 3), HO− showed an increase in NDI [whole hippocampus: *t*(82) = 5.049, *P* < 0.001, Holm-Bonferroni corrected, partial η^2^ = 0.239; parahippocampus: *t*(82) = 4.447, *P* < 0.001, Holm-Bonferroni corrected, partial η^2^ = 0.196; ERC: *t*(82) = 3.338, *P* = 0.009, Holm-Bonferroni corrected, partial η^2^ = 0.121; PRC: *t*(82) = 2.970, *P* = 0.016, Holm-Bonferroni corrected, partial η^2^ = 0.098], ODI [whole hippocampus: *t*(82) = 6.581, *P* < 0.001, Holm-Bonferroni corrected, partial η^2^ = 0.348; ERC: *t*(82) = 3.291, *P* = 0.009, Holm-Bonferroni corrected, partial η^2^ = 0.118], and V_*iso*_ [whole hippocampus: *t*(82) = 6.939, *P* < 0.001, Holm-Bonferroni corrected, partial η^2^ = 0.373; parahippocampus: *t*(82) = 5.402, *P* < 0.001, Holm-Bonferroni corrected, partial η^2^ = 0.265; ERC: *t*(82) = 5.742, *P* < 0.001, Holm-Bonferroni corrected, partial η^2^ = 0.289; PRC: *t*(82) = 4.375, *P* < 0.001, Holm-Bonferroni corrected, partial η^2^ = 0.191] as compared to HY.

### Effects of asymptomatic Alzheimer’s disease pathology

Significantly increased eTIV [*t*(68) = 2.956, *P* = 0.026, Holm-Bonferroni corrected, partial η^2^ = 0.114] was observed in Ho+ as compared to HO− (Table 1). As expected, no statistically significant differences in neuropsychological performance were found between HO− and Ho+ groups (Table 2).

Shape analysis using FSL FIRST showed that compared to HO−, regional atrophy (*P* < 0.05, FWE corrected using TFCE) was found in Ho+ on the inferior side of the hippocampus, which localized primarily to the subiculum and CA1-3 (Figures 2B,C). No significant differences in functional connectivity (Supplementary Figures 4, 6) and diffusion metrics (Supplementary Figures 5, 7) were observed between HO− and Ho+.

### Correlations between imaging metrics and neuropsychological performance

**FIGURE 5:**
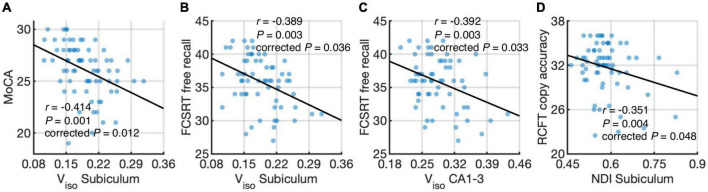
Correlations of hippocampal subfield connectivity and diffusion metrics with neuropsychological performance: volume fraction of isotropic water diffusion (V_*iso*_) in the subiculum vs. the Montreal Cognitive Assessment (MoCA) score **(A)** V_*iso*_ in the subiculum vs. Free and Cued Selective Reminding Test (FCSRT) free recall score **(B)** V_*iso*_ in CA1-3 vs. FCSRT free recall score **(C)** and neurite density index (NDI) in the subiculum vs. Rey Complex Figure Test (RCFT) copy accuracy score **(D)**. The associations were evaluated using partial correlation with age, sex, normalized whole hippocampal volume, and total intracranial volume as covariates. *P*-values were adjusted for multiple comparisons using Holm-Bonferroni correction. The fitting lines are also shown to indicate trends.

Figure 5 shows associations between hippocampal subfield imaging metrics and neuropsychological assessments using data from the elderly groups. We found differential correlations of hippocampal subfields with neuropsychological assessments. V_*iso*_ quantifies water component of local tissue and reflects microscopic atrophy. V_*iso*_ in the subiculum was found to negatively correlate with the MoCA, a measure of global cognition (*r* = −0.414, *P* = 0.012, Holm-Bonferroni corrected), and FCSRT free recall, a measure of memory function (*r* = −0.389, *P* = 0.036, Holm-Bonferroni corrected). Also, V_*iso*_ in CA1-3 correlated negatively with FCSRT free recall (*r* = −0.392, *P* = 0.033, Holm-Bonferroni corrected). NDI reflects neurite density and its value in the subiculum correlated negatively with RCFT copy accuracy score, a measure of visuospatial function (*r* = −0.351, *P* = 0.048, Holm-Bonferroni corrected).

For medial temporal lobe regions (Supplementary Figure 8), negative correlations were found between V_*iso*_ in the whole hippocampus and FCSRT free recall (*r* = −0.415, *P* = 0.016, Holm-Bonferroni corrected) and between V_*iso*_ in the parahippocampus and FCSRT free recall (*r* = −0.390, *P* = 0.045, Holm-Bonferroni corrected).

### Correlations between cerebral spinal fluid biomarkers and imaging metrics

**FIGURE 6:**
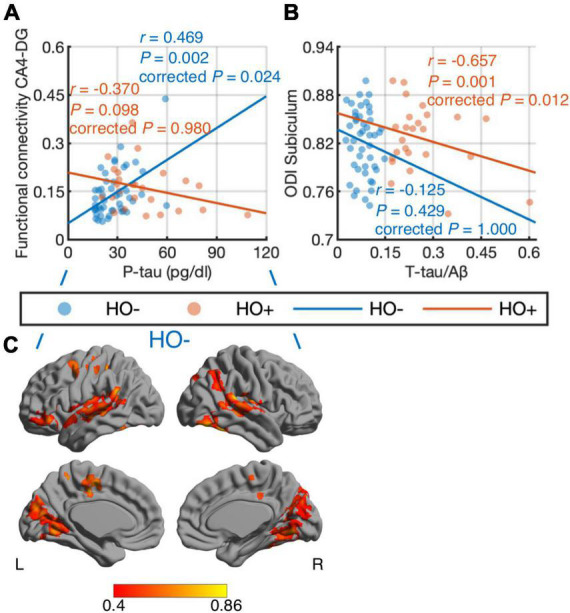
Correlations of CSF biomarker measurements with hippocampal subfield connectivity and diffusion metrics: tau phosphorylated at threonine 181 (P-tau) vs. functional connectivity in CA4-DG network **(A,C)** and total tau (T-tau)/amyloid-β 1-42 (Aβ) ratio vs. orientation dispersion index (ODI) in the subiculum **(B)**. The associations were evaluated using partial correlation with age, sex, normalized whole hippocampal volume, and total intracranial volume as covariates. *P*-values were adjusted for multiple comparisons using Holm-Bonferroni correction. Blue and orange dots represent healthy older adults with negative CSF biomarker status (HO−) and positive CSF biomarker status (Ho+), respectively. The fitting lines are also shown to indicate trends for HO− (blue lines) and Ho+ (orange lines). In voxel-wise analysis in HO− **(C)**, the results were corrected using threshold-free cluster enhancement where family-wise error rate was controlled at 0.05.

Differential correlations of hippocampal subfields with CSF biomarkers were found. When combining HO− and Ho+, no significant correlations were found between CSF biomarker measurements and imaging metrics. We further performed correlation analysis in HO− and Ho+ groups separately. With age, sex, normalized whole hippocampal volume and eTIV as covariates, partial correlation (Figures 6A,B) showed positive correlation between CA4-DG network connectivity and P-tau in HO− (*r* = 0.469, *P* = 0.024, Holm-Bonferroni corrected), as well as negative correlations between ODI, an index of fiber dispersion, in the subiculum and T-tau/Aβ ratio in Ho+ (*r* = −0.657, *P* = 0.012, Holm-Bonferroni corrected). For CA4-DG network, voxel-wise analysis (Figure 6C) further showed significant correlations of P-tau with functional connectivity of CA4-DG to brain regions in the frontal pole, frontal orbital cortex, temporal pole, superior temporal gyrus, middle temporal gyrus, posterior supramarginal gyrus, lingual gyrus, precuneus, intracalcarine cortex, precentral gyrus, juxtapositional lobule cortex, and inferior lateral occipital cortex (*P* < 0.05, FWE corrected using TFCE).

## Discussion

To our best knowledge, this represents the first multimodal MRI study reporting the effects of normal and pathological aging on morphometry, intrinsic functional connectivity, and tissue microstructure of hippocampal subfields in cognitively normal adults with known biomarkers of AD pathology. Our results revealed regional atrophy, reduced functional connectivity, and increased microstructural metrics NDI, ODI and V_*iso*_ in all hippocampal subfields with normal aging. As Aβ and pathologic tau accumulated in cognitively normal adults, regional atrophy was observed primarily in the subiculum and CA1-3. Microstructural metrics from diffusion MRI were significantly associated with neuropsychological assessments. CSF tau showed close correlation with synaptic and microstructural measures derived from resting-state functional MRI and diffusion MRI.

A large body of resting-state functional MRI evidence demonstrates that the most common pattern of age-related changes is the reduction of connectivity in brain functional networks, although discrepant results have been reported (Ferreira and Busatto, 2013) due to differences in acquisition and analysis methods. In all the hippocampal subfield networks examined in the current study, decreased functional connectivity was extensively distributed over the hippocampal network in the aging brain. The most pronounced decrements were found in CA1-3 and CA4-DG, consistent with our morphometric results and previous neuroimaging studies measuring volume changes of hippocampal subfields (Mueller et al., 2007; Mueller et al., 2008; Mueller and Weiner, 2009; Adler et al., 2018; Uribe et al., 2018; Nadal et al., 2020). The age-related connectivity disruptions are tentatively attributed to diminished white matter integrity, dopaminergic dysfunction and Aβ deposition (Ferreira and Busatto, 2013). Interestingly, it is hypothesized that the relationship between functional connectivity and Aβ deposition is bidirectional, i.e., brain regions with high resting-state functional connectivity, such as the default mode network, are prone to Aβ accumulation; and Aβ deposition results in connectivity deficits (Bero et al., 2012). It has also been suggested that tau-related insults can lead to de-differentiation of cortical functional specificity (Maass et al., 2019), and our results can be interpreted to indicate that hippocampal deteriorations might play a role in such de-differentiation.

In addition to functional connectivity alterations, our results of diffusion MRI demonstrate changes in tissue microstructure across all hippocampal subfields with normal aging. Compared to HY, HO− showed elevated NDI, reflecting age-related increase in neurite density of the hippocampus. As an indicator of dendritic complexity, ODI in the hippocampal subfields was higher in HO− than that in HY, in accordance with a previous NODDI study (Nazeri et al., 2015). The changes of NDI and ODI suggest dendritic growth and extension with advancing age, which are regarded as a compensatory response to partial deafferentation (Buell and Coleman, 1979; Pyapali and Turner, 1996). Also, in agreement with a previous neuroimaging study (Merluzzi et al., 2016), we observed increased V_*iso*_ in the hippocampus, plausibly indicating an early sign of hippocampal atrophy. Although it is indicated that NODDI measures do not accurately reflect neuritic dispersion (Lampinen et al., 2017), our results demonstrated group differences between HO− and HY as well as correlations of NODDI metrics with CSF biomarkers and neuropsychological assessments, suggesting that NODDI measures could be used as potential biomarkers for normal aging and AD regardless their underlying biological underpinning.

The hippocampus is a key structure in the development of AD, in which abnormality occurs early in the disease process (Braak and Braak, 1995; Braak et al., 2006). Compared to PET images and CSF biomarker measurements, MRI-based biomarkers are non-invasive, and are relatively inexpensive for early diagnosis and prediction of AD disease progression. In this study, the subiculum and CA1-3 showed the most pronounced atrophy and the most pervasive correlations of imaging measures with neuropsychological assessments, providing support to previous findings that the subiculum and CA1 demonstrate the earliest sensitivity to AD pathology among the hippocampal subfields (Braak and Braak, 1991; Carlesimo et al., 2015). Furthermore, we observed that such hippocampal atrophy appeared to be asymmetric with reduced volume on the left hippocampus localized to the head region and reduced volume on the right hippocampus localized to the tail region. There were previous studies reporting asymmetry in hippocampal volumes in MCI and AD dementia (Shi et al., 2009; Minkova et al., 2017; Sarica et al., 2018). Our results of asymmetric hippocampal atrophy in the asymptomatic stage of AD appear to be consistent with these reports, although further validations in larger studies are needed.

Only V_iso_ was found to significantly correlate with memory functioning when studying the whole hippocampus. However, when splitting the hippocampus into subfields, we observed V_iso_ and NDI correlated with a wider range of cognitive functions, suggesting that the differential correlations between hippocampal subfields and neuropsychological performance would be missed if hippocampal subfields were combined. In concordance with a previous volumetric study in a population-based cohort (Evans et al., 2018), we observed that a broad range of cognitive functions were associated with imaging measures of hippocampal subfields, whereas hippocampal deficits in the subclinical stage of dementia are generally believed to contribute to memory decline (Apostolova et al., 2006; den Heijer et al., 2010). This finding suggests that besides memory function, a wide range of cognitive alterations would accompany the hippocampal changes in the early stages of AD cascade. Moreover, we found that the subiculum and CA1-3 demonstrated the most robust correlations between imaging measures and neuropsychological performance. This could be associated with subjective cognitive impairment among the cognitively normal subjects, where individuals complain about memory and other cognitive difficulties without clinical diagnosis of cognitive impairment. Subjective cognitive impairment in adults with normal neuropsychological performance may represent the first clinical sign of risk in developing to dementia subsequently (Jessen et al., 2014). It has been found to correlate with focal atrophy in the hippocampus and its subfields primarily including the subiculum and CA1 (Van der Flier et al., 2004; Perrotin et al., 2015). Further studies are needed with respect to early changes in hippocampal subfields and subjective cognitive impairment.

Compared to CSF Aβ, tau may correlate better with hippocampal atrophy, as suggested by previous volumetric studies in symptomatic stages of AD (Henneman et al., 2009; Apostolova et al., 2010; de Souza et al., 2012). Our results further corroborate the associations of CSF tau with hippocampal abnormality. Furthermore, seed-competent tau throughout the Braak tau pathway has been shown to precede any overt regional tau pathology (DeVos et al., 2018) along ERC, to hippocampus, posterior parahippocampal gyrus, anterior cingulate cortex, visual association region, and eventually primary visual cortex (Braak and Braak, 1995; Braak et al., 2006). It has been reported that tau aggregates propagate from ERC to synaptically connected DG in a mouse model of early AD (de Calignon et al., 2012; Pickett et al., 2017). Intriguingly, we observed that along the Braak tau pathway, CSF tau correlated with functional connectivity in CA4-DG network in cognitively normal adults with negative AD pathology, and then with ODI in the subiculum in cognitively normal adults with positive AD pathology. This finding offers support that tau pathology plays an important role in pre-symptomatic AD.

It may be argued that there are partial volume effects associated with our analysis. We used 1 mm isotropic resolution for the segmentation of the hippocampal subfields, which might not be optimal for defining hippocampal subfield ROIs (Wisse et al., 2021). However, FreeSurfer 6.0-based segmentation of hippocampal subfields from T_1_-weighted images with standard resolution (1 mm isotropic) has shown the ability to detect expected effects of AD (de Flores et al., 2015; Iglesias et al., 2015; Sämann et al., 2022). For diffusion MRI, expected biological effects were observed in previous studies on normal aging, AD pathology, and epilepsy using spatial resolution similar to the current study (Pereira et al., 2014; Wolf et al., 2015; Goubran et al., 2016; Carlson et al., 2021). Moreover, the top and bottom 5% data were excluded in calculating regional mean values to reduce partial volume effects. For functional MRI, we aimed to evaluate hippocampal subfield connectivity to the rest of the brain. To facilitate this, functional MRI data were acquired at a spatial resolution of 1.5 mm isotropic, which is much better than typical resting-state functional MRI. The temporal signals of subfield ROIs were obtained before spatial smoothing to reduce the partial volume effects in extracting seed temporal series. In addition, although the hippocampal subfield networks are visually similar to each other (Vos de Wael et al., 2018), the differences between the hippocampal subfield connectivity have been reported in previous resting-state functional MRI studies using lower spatial resolution than the present study (de Flores et al., 2017; Vos de Wael et al., 2018). In the current study, we also observed differences between the hippocampal subfield networks. These results indicated that the hippocampal subfield connectivity was not dominated by the partial volume effects. Nonetheless, partial volume effects are still expected to exist. The development of high-resolution scan technique combined with the benefits of ultrahigh field such as 7-Tesla could further mitigate partial volume effects.

Several issues remain to be addressed in the future. First, DG is mostly inside the structure, and its morphometric changes could not be fully evaluated by shape analysis. Studies combining higher spatial resolution with volumetric analysis are needed to further investigate morphometric changes of hippocampal subfields. Second, more female participants were recruited in this study, especially in the Ho+. Our findings were thus skewed to females. Data with more balanced sample distribution between males and females are required in future work. Third, this work included cross-sectional data in the analysis. Examining longitudinal changes in hippocampal subfield connectivity and microstructure, especially among asymptomatic elderly with positive CSF biomarker status, remains the subject of future studies. The collection of follow-up data is currently underway as part of our EHBS. Finally, CSF biomarkers were used to measure amyloid-β and tau in the current study. Further studies with amyloid and tau PET scans may provide complementary information on spatial specificity for the correlations of hippocampal subfield connectivity and microstructure with regional deposition levels of amyloid and tau.

In summary, the morphometry, intrinsic functional connectivity, and tissue microstructure in all hippocampal subfields are disrupted with normal aging, while the subiculum and CA1-3 show the greatest vulnerability to asymptomatic AD pathology. Microstructural metrics from diffusion MRI are associated with neuropsychological assessments. Tau, rather than amyloid-β, intimately correlates with synaptic and microstructural measures in hippocampal subfields. Hippocampal subfield connectivity and microstructural measures could be promising imaging markers for early detection and prognosis of AD.

## Data availability statement

Requests to access the raw data can be made to the corresponding authors by qualified investigators and a data use agreement might be required per institutional policies of Emory University.

## Ethics statement

The studies involving human participants were reviewed and approved by the Emory University School of Medicine Institutional Review Board. The patients/participants provided their written informed consent to participate in this study.

## Author contributions

JW, DL, FG, AL, JL, and DQ conceptualized the study and interpreted the data. JW, SS, QL, AH-B, and AB performed the acquisition of the data. JW, SS, QL, JS, and BR performed the analysis and quality control of the image data. JW and DQ wrote the manuscript. All authors contributed to the reviewing and editing the manuscript.

## Footnotes

1
https://github.com/AthenaEPI/dmipy
