# Supplementary material for: Multimodal magnetic resonance imaging reveals distinct sensitivity of hippocampal subfields in asymptomatic stage of Alzheimer’s disease
Source: Front Aging Neurosci. 2022 Aug 12;14:901140. doi: 10.3389/fnagi.2022.901140 (PMC9413400; doi:10.3389/fnagi.2022.901140)
Supplement: Supplementary file 1 [file Table_1.DOCX]

Supplementary Table 1. Functional connectivity and microstructural measures of hippocampal subfields in a group of healthy young adults

|  | Functional connectivity | NDI | ODI | V_iso_ |
| --- | --- | --- | --- | --- |
| Hippocampal subfields |  |  |  |  |
| Subiculum | 0.254 ± 0.127 | 0.536 ± 0.030 | 0.759 ± 0.050 | 0.146 ± 0.028 |
| CA1-3 | 0.222 ± 0.112 | 0.560 ± 0.033 | 0.701 ± 0.033 | 0.235 ± 0.034 |
| CA4-DG | 0.242 ± 0.117 | 0.492 ± 0.037 | 0.716 ± 0.037 | 0.145 ± 0.034 |
| Subiculum vs. CA1-3^1^ |  |  |  |  |
| *t* statistic | 4.714 | -4.812 | 8.225 | -17.426 |
| *P* value | < 0.001^***^ | < 0.001^***^ | < 0.001^***^ | < 0.001^***^ |
| *η*^2^ | 0.363 | 0.373 | 0.634 | 0.886 |
| Subiculum vs. CA4-DG^1^ |  |  |  |  |
| *t* statistic | 1.075 | 9.855 | 4.954 | 0.46 |
| *P* value | 0.578 | < 0.001^***^ | < 0.001^***^ | 0.648 |
| *η*^2^ | 0.029 | 0.713 | 0.386 | 0.005 |
| CA1-3 vs. CA4-DG^1^ |  |  |  |  |
| *t* statistic | -1.625 | 18.615 | -2.700 | 21.907 |
| *P* value | 0.337 | < 0.001^***^ | 0.041^*^ | < 0.001^***^ |
| *η*^2^ | 0.063 | 0.899 | 0.157 | 0.925 |

NDI= neurite density index; ODI = orientation dispersion index; V_iso_ = volume fraction of isotropic water diffusion.

^1^Differences between hippocampal subfields were evaluated using paired *t*-tests.

Significant at ^*^*P* < 0.05 and ^***^*P* < 0.001, Holm-Bonferroni corrected.
