# Supplementary material for: Multimodal magnetic resonance imaging reveals distinct sensitivity of hippocampal subfields in asymptomatic stage of Alzheimer’s disease
Source: Front Aging Neurosci. 2022 Aug 12;14:901140. doi: 10.3389/fnagi.2022.901140 (PMC9413400; doi:10.3389/fnagi.2022.901140)
Supplement: Supplementary file 2 [file Image_1.PDF]

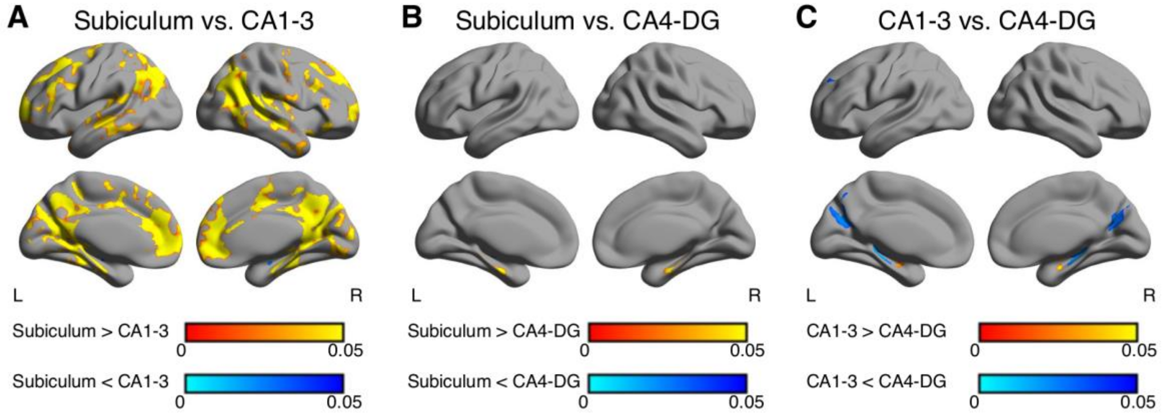

Supplementary Figure 1. Differences between hippocampal subfield networks in a group of healthy young adults. *P*-values were determined using paired t-tests, and corrected using threshold-free cluster enhancement where family-wise error rate was controlled at 0.05.
