# Supplementary material for: Multimodal magnetic resonance imaging reveals distinct sensitivity of hippocampal subfields in asymptomatic stage of Alzheimer’s disease
Source: Front Aging Neurosci. 2022 Aug 12;14:901140. doi: 10.3389/fnagi.2022.901140 (PMC9413400; doi:10.3389/fnagi.2022.901140)
Supplement: Supplementary file 5 [file Image_4.PDF]

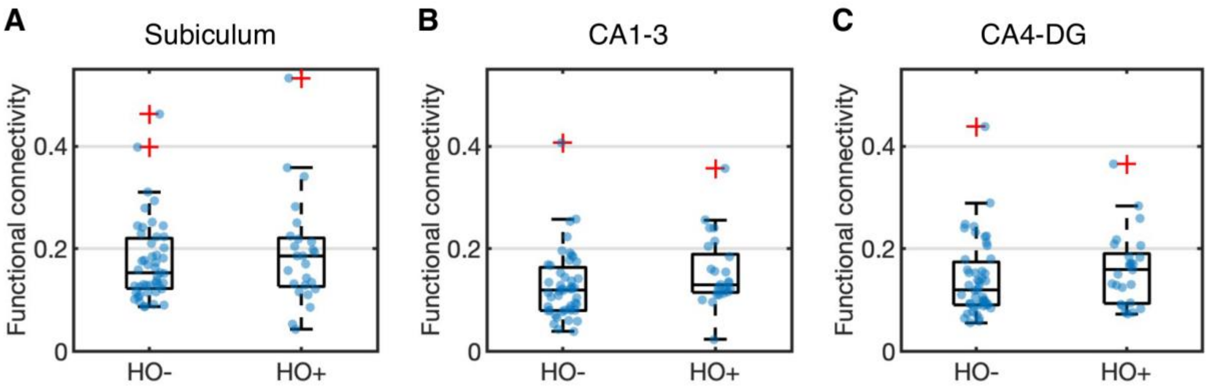

Supplementary Figure 4. Group differences of functional connectivity in the subiculum (A), CA1-3 (B) and CA4-DG (C) networks between healthy older adults with negative CSF biomarker status (HO-) and positive CSF biomarker status (HO+). Box plots show the median, quartiles and whiskers that represent 1.5 × the interquartile range. *P*-values were determined using general linear models with age, sex, normalized whole hippocampal volume, and total intracranial volume as covariates, and adjusted for multiple comparisons using Holm-Bonferroni correction.
