# Supplementary material for: Multimodal magnetic resonance imaging reveals distinct sensitivity of hippocampal subfields in asymptomatic stage of Alzheimer’s disease
Source: Front Aging Neurosci. 2022 Aug 12;14:901140. doi: 10.3389/fnagi.2022.901140 (PMC9413400; doi:10.3389/fnagi.2022.901140)
Supplement: Supplementary file 6 [file Image_5.PDF]

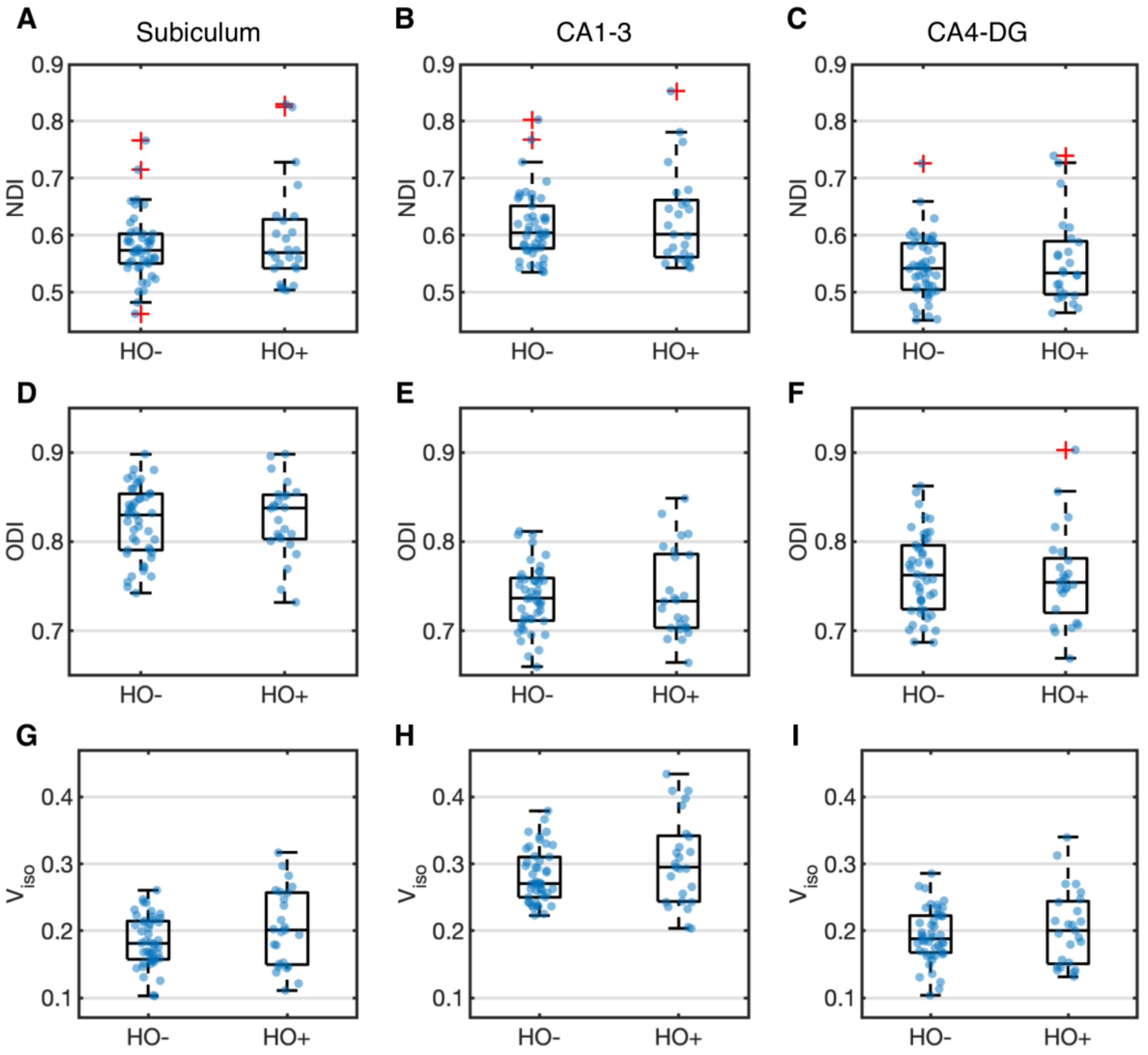

Supplementary Figure 5. Group differences of neurite density index (NDI) (A, B and C), orientation dispersion index (ODI) (D, E and F) and volume fraction of isotropic water diffusion (V_{iso}) (G, H and I) in the subiculum (A, D and G), CA1-3 (B, E and H) and CA4-DG (C, F and I) between healthy older adults with negative CSF biomarker status (HO-) and positive CSF biomarker status (HO+). Box plots show the median, quartiles and whiskers that represent 1.5 × the interquartile range. *P*-values were determined using general linear models with age, sex, normalized whole hippocampal volume, and total intracranial volume as covariates, and adjusted for multiple comparisons using Holm-Bonferroni correction.
