# Supplementary material for: Multimodal magnetic resonance imaging reveals distinct sensitivity of hippocampal subfields in asymptomatic stage of Alzheimer’s disease
Source: Front Aging Neurosci. 2022 Aug 12;14:901140. doi: 10.3389/fnagi.2022.901140 (PMC9413400; doi:10.3389/fnagi.2022.901140)
Supplement: Supplementary file 7 [file Image_6.PDF]

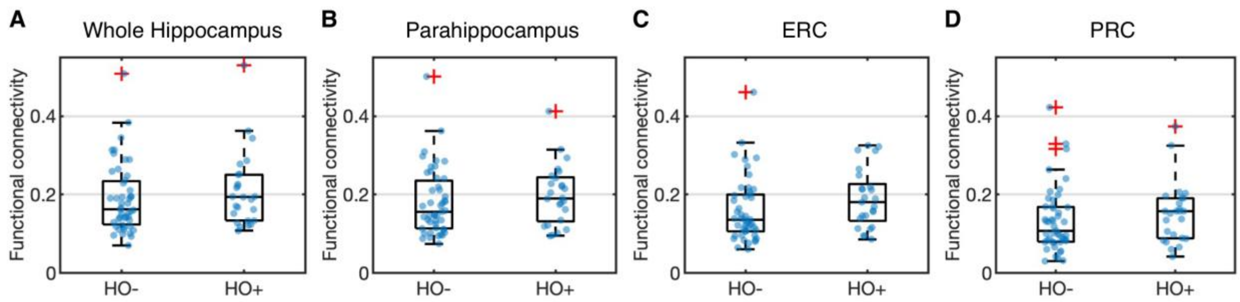


**Supplementary Figure 6.** Group differences of functional connectivity in the whole hippocampus (A), parahippocampus (B), entorhinal cortex (ERC) (C), perirhinal cortex (PRC) (D) networks between healthy older adults with negative CSF biomarker status (HO-) and positive CSF biomarker status (HO+). Box plots show the median, quartiles and whiskers that represent 1.5 × the interquartile range. *P*-values were determined using general linear models with age, sex, normalized volume of corresponding brain region, and total intracranial volume as covariates, and adjusted for multiple comparisons using Holm-Bonferroni correction.
