# Supplementary material for: Multimodal magnetic resonance imaging reveals distinct sensitivity of hippocampal subfields in asymptomatic stage of Alzheimer’s disease
Source: Front Aging Neurosci. 2022 Aug 12;14:901140. doi: 10.3389/fnagi.2022.901140 (PMC9413400; doi:10.3389/fnagi.2022.901140)
Supplement: Supplementary file 8 [file Image_7.PDF]

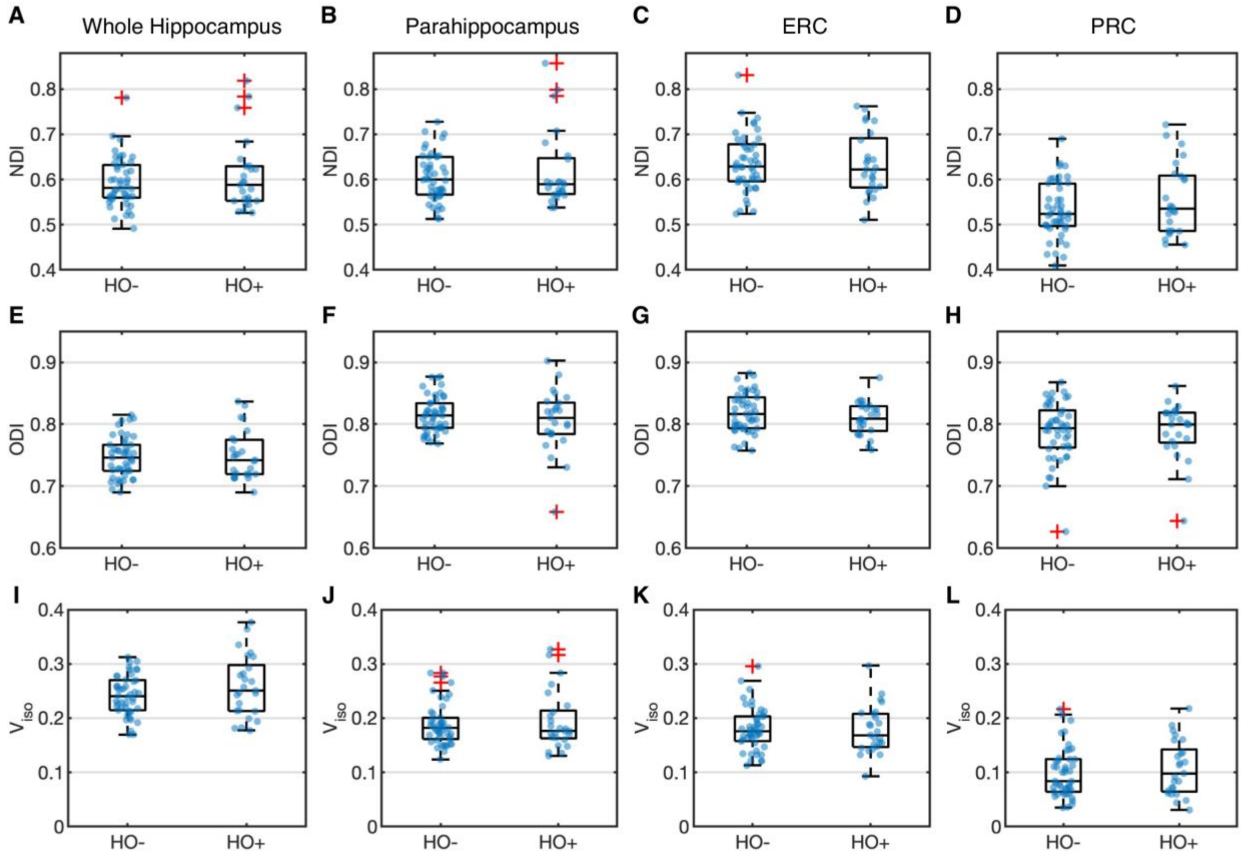

Supplementary Figure 7. Group differences of neurite density index (NDI) (A, B, C and D), orientation dispersion index (ODI) (E, F, G and H) and volume fraction of isotropic water diffusion (V_{iso}) (I, J, K and L) in the whole hippocampus (A, E and I), parahippocampus (B, F and J), entorhinal cortex (ERC) (C, G and K), perirhinal cortex (PRC) (D, H and L) between healthy older adults with negative CSF biomarker status (HO-) and positive CSF biomarker status (HO+). Box plots show the median, quartiles and whiskers that represent 1.5 × the interquartile range. *P*-values were determined using general linear models with age, sex, normalized volume of corresponding brain region, and total intracranial volume as covariates, and adjusted for multiple comparisons using Holm-Bonferroni correction.
