# Supplementary material for: Multimodal magnetic resonance imaging reveals distinct sensitivity of hippocampal subfields in asymptomatic stage of Alzheimer’s disease
Source: Front Aging Neurosci. 2022 Aug 12;14:901140. doi: 10.3389/fnagi.2022.901140 (PMC9413400; doi:10.3389/fnagi.2022.901140)
Supplement: Supplementary file 9 [file Image_8.pdf]

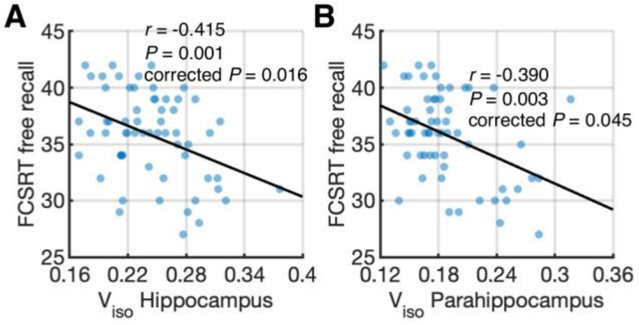

Supplementary Figure 8. Correlations of functional connectivity and diffusion metrics with neuropsychological performance: V_{iso} in the whole hippocampus vs. Free and Cued Selective Reminding Test (FCSRT) free recall score (A) and V_{iso} in the parahippocampus vs. FCSRT free recall score (B). The associations were evaluated using partial correlation with age, sex, normalized volume of corresponding brain region, and total intracranial volume as covariates. *P*-values were adjusted for multiple comparisons using Holm-Bonferroni correction. The fitting lines are also shown to indicate trends.
